# Neurological growth and development of children asymptomatic at birth whose mothers had Zika during pregnancy

**DOI:** 10.1590/0037-8682-0180-2020

**Published:** 2021-02-10

**Authors:** Ana Maria Peixoto Cabral Maia, Camila de Sousa Lins Azevedo, Rhaquel de Moraes Alves Barbosa de Oliveira, Francisca Kalline Almeida Barreto, Adilina Soares Romeiro Rodrigues, Adriana Rocha Simião, Ileana Pitombeira Gomes, Erlane Marques Ribeiro, Luciano Pamplona de Góes Cavalcanti

**Affiliations:** 1 Universidade Federal do Ceará, Programa de Pós-graduação em Saúde Coletiva, Fortaleza, CE, Brasil.; 2 Secretaria de Saúde do Município de Fortaleza, Fortaleza, CE, Brasil.; 3 Secretaria de Saúde do Estado do Ceará, Fortaleza, CE, Brasil.; 4 Hospital Infantil Albert Sabin, Fortaleza, CE, Brasil.; 5 Centro Universitário Christus, Faculdade de Medicina, Fortaleza, CE, Brasil.; 6 Universidade Federal do Ceará, Programa de Pós-graduação em Patologia, Fortaleza, CE, Brasil.

**Keywords:** Zika virus, Microcephaly, Child development

## Abstract

**INTRODUCTION:**

Newborn who had Zika vírus but did not show microcephaly at birth may have neuropsychomotor development problems. We aimed to evaluate the developmental and anthropometric milestones of asymptomatic children whose mothers had Zika during pregnancy in Northeastern Brazil in 2015 and 2016.

**METHODS:**

We conducted a descriptive cross-sectional case series study of children in Fortaleza born without microcephaly whose mothers had Zika during pregnancy. Home visits were undertaken to evaluate the developmental milestones and gather anthropometric data of the children and to conduct semi-structured interviews with the mothers to identify their socioeconomic and gestational profiles and assess the newborns after birth.

**RESULTS:**

In total, 30 cases were identified. Of these, 17 children and their mothers participated in the study. The median age of the mothers at the time of delivery was 26 years. All were symptomatic, and TORCH was negative. At the time of the home visit, all had growth profiles suitable for their age. However, nearly all children (15/17, 88.2%) presented at least one developmental delay, considering their age group.

**CONCLUSIONS:**

There were late changes in the neuropsychomotor development of children born to mothers who had Zika during pregnancy, suggesting the need for specialized medical follow-ups.

## INTRODUCTION

The Zika virus (ZIKV) is a flavivirus that was first isolated in 1947 from the Zika forest in Uganda. It is mainly transmitted by the *Aedes aegypti* mosquito, and the first human infections were recorded in Southeast Asia and Sub-Saharan Africa^1^ ^,^ ^2^ ^,^ ^3^.

In October 2015, there was an unexpected increase in the births of children with microcephaly in the State of Pernambuco and later in other states in the Northeast region of Brazil. This coincided with the confirmation of the autochthonous transmission of ZIKV in Brazil in April of the same year, a fact that led scientists to establish an association between the two events^4^.

In addition to congenital microcephaly, other neurological and musculoskeletal manifestations may be present in infants who acquire ZIKV during gestation. This clinical condition was characterized as the congenital Zika syndrome (CZS)^5^.

Some children with CZS are born without microcephaly and appear asymptomatic but present with neurological changes that can be evidenced only by neuroimaging. Therefore, some children may not have been diagnosed at birth owing to their normal head circumferences (HC)^6^.

Studies suggest that children exposed to ZIKV during gestation, but born without microcephaly or other noticeable changes, may develop late CZS-related manifestations and present with developmental abnormalities such as hypotonia, hypertonia, signs of ataxia, dyskinesia, and irritability^7^. ZIKV has been reported in children born with CZS, although few studies have reported on newborns who were asymptomatic at birth but whose mothers had ZIKV during pregnancy. These newborns may present with neuropsychomotor developmental problems that become apparent later. Therefore, it is important to monitor these children and their developmental milestones during the first few years of life, provided there are records of changes in this development^5^ ^,^ ^8^.

Thus, the current study evaluated cases of pregnant women diagnosed with Zika with laboratory confirmation whose children were born with neither microcephaly nor musculoskeletal malformations. We also aimed to evaluate the anthropometric profiles and developmental milestones of children born without microcephaly whose mothers had Zika during gestation in the city of Fortaleza, Ceará, in 2015 and 2016.

## METHODS

### Ethical aspects

The ethical principles set forth in Resolution 466/12 of the National Health Council were followed. All those responsible signed the free and informed consent form authorizing the completion of the questionnaire and allowing evaluation of the children. The research was approved by the Research Ethics Committee through CAAE No. 75393417.4.0000.5054.

### Type and design of the study

A cross-sectional case series study was conducted among the residents of Fortaleza in children asymptomatic at birth whose mothers had laboratory confirmed ZIKV infections during pregnancy.

### Data collection

Initially, all the reported cases of pregnant women with suspected ZIKV infections in the National Notifiable Diseases Information System (SINAN) between 2015 and 2016 were investigated, and those who did not have laboratory confirmations were excluded. Subsequently, children who did not present with microcephaly at birth were selected through the Live Birth Information System (SINASC) from the field of the declaration of live births (DNV), which provided information of the malformations presented by the newborns at birth. We then identified all pregnant women who presented with laboratory-confirmed ZIKV infections and gave birth to children without microcephaly.

The HCs were evaluated using the INTERGROWTH-21st curve^9^ to confirm that none of the children had HCs at birth that were below 2 standard deviations for their sex and gestational age.

For data collection, the mothers were contacted via telephone to schedule home visits. When the mother's telephone number was unavailable, the visit was undertaken during business hours at the address provided on the notification form. In cases where the families were not available at their residences, the cases were classified as missing after three attempts were made on alternate days and at different times. Additionally, the children whose families were living in the interior of the State of Ceará were excluded. During the home visits, the mothers were interviewed, and the children were clinically evaluated to assess the anthropometric data and developmental milestones according to the child's health booklet.

### Data collection instrument

A semi-structured questionnaire was developed by the researchers and administered during the interviews with the mothers. This instrument was divided into eight sections, including socioeconomic data on the mother, her obstetric and gynecological background, maternal pathological antecedents, prenatal and birth data, and complementary examinations.

We also used the Child Health Manual developed by the Brazilian Ministry of Health for anthropometric and neurological evaluation of the child based on the developmental milestones contained in the booklet. For the anthropometric evaluation of the children, measurements such as weight (g), height (cm), HC (cm), and thoracic perimeter (cm) were taken. For this evaluation, the growth curve of the World Health Organization that was available in the child health handbook was used^10^. 

The child's weight was taken using a platform-type digital scale, placed on a flat surface. During this evaluation, the child was barefoot, wearing only a diaper or an underwear. For children who did not stand on the scale, the weights of the mother and child were evaluated together, after which the mother's weight was subtracted.

The height of the children was measured, in centimeters, using an anthropometric ruler with the child lying down on a flat surface.

The cephalic perimeter was evaluated by measuring with a non-retractable tape measure positioned over the occipital prominence and over the arches of the ears. The tape was placed around the frontal bone over the supra orbital groove, passing on the same level on each side and placed on the occipital prominence. To assess the child's development, the developmental milestones for the specific age available in the child's health handbook were used, as recommended by the Ministry of Health of Brazil^10^.

## RESULTS

**FIGURE 1:**
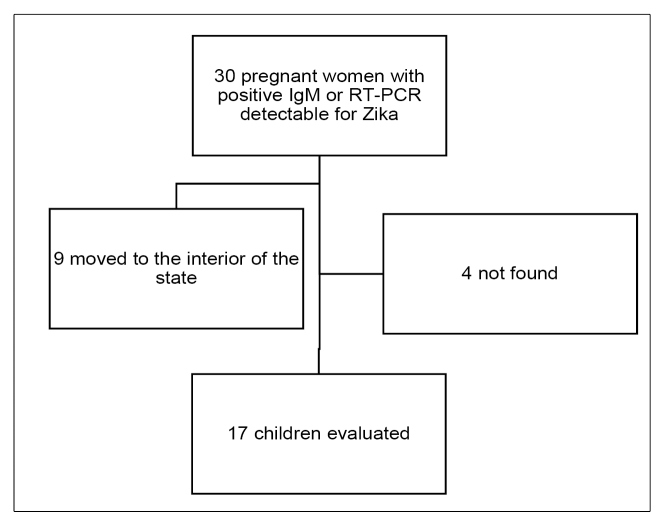
Flow chart of children eligible for the study.

We identified 30 children who met the inclusion criteria for the study. After telephonic contact or visits to the residences, four mothers were not located at home and nine had moved to the interior of the state of Ceará. Therefore, only 17 children were included in the study (Figure 1).

### Characteristics of the pregnant women

The median age of the women at the time of delivery was 26 years (range: 20-29 years) (12/17, 70.6%), with only one-third of them having completed high school (7/17, 41.2%). The average age of the 13 women who did not participate in the study was 24 years (range, 17-34 years).

A total of 12/17 (70.6%) declared that they were white, 7/17 (41.2%) were primiparous, 5/17 (29.4%) had a history of previous abortion, 7/17 (41.2%) were married, and 8/17 (47.1%) were housewives. The average income was one minimum wage (954.00 BRL). Among the women who were housewives, 3/8 (37.5%) had quit their jobs to take care of their children (Table 1). 

**TABLE 1:** Maternal socioeconomic profile.

Variables	N=17	%
**Age (years)**		
20-29	12	70.6
30-9	4	23.5
>40	1	5.9
**Color/race**		
White	12	70.6
Brown	5	29.4
Schooling*		
Fundamental	6	35.3
Medium	7	41.2
Higher	4	23,5
**Marital status**		
Single	4	23.5
Married	7	41.2
Stable union	6	35.3
**Occupation**		
Housewife	8	47.0
Autonomous	5	29.4
CLL (Consolidated Labor Law)	2	11.8
Student	1	5.9
Public worker	1	5.9
**Family income**		
<1 minimum wage	11	64.7
1-3 times the minimum wage	5	29.4
≥4 times the minimum wage	1	5.9

Legend: *fundamental ( ≤ 7 years), medium (7-14 years), and higher (> 14 years).

All the mothers presented with the symptomatic form of the disease: a skin rash was observed in 100% of the pregnant women. A total of 16 (94.1%) patients presented with pruritus. These symptoms predominately occurred in the second trimester (8/17, 47.1%), followed by the third (6/17, 35.3%) and the first (3/17, 17.6%) trimesters. A majority (10/17, 58.8%) reported not using medication at the onset of symptoms (Table 2).

**TABLE 2:** Exposure to ZIKV during pregnancy and the occurrence of other infections.

Variables	N=17	%
**Skin rash**		
Yes	17	100.0
No	-	
**Itch**		
Yes	16	94.1
No	1	5.9
**Fever**		
Yes	9	52.9
No	8	47.1
**Gestational period at the time of infection with ZIKV**		
<13 weeks (1st trimester)	3	17.6
14-28 (2nd trimester)	8	47.1
>29 (3rd trimester)	6	35.3
**Use of medication during infection**		
Yes	7	41.2
No	10	58.8
**Other signs and symptoms during pregnancy suggestive of infection**		
Yes	3	17.6
No	14	82.4
Exposure to chemical substances*		
Yes	3	17.6
No	14	82.4

*One mother reported contact with insecticide.

All the mothers had undergone at least five prenatal consultations, and 13/17 (76.5%) had undergone more than seven visits. All TORCH serologies (Toxoplasmosis, Rubella, Cytomegalovirus, and Herpes simplex virus) were negative. Five pregnant women reported complications such as gestational diabetes (2), pre-eclampsia (1), anemia (1), and pyelonephritis (1). Only one patient had previously been vaccinated against yellow fever.

### Characteristics of the children

There was no significant predominance of sex (9/8 M/F). The highest number of births was recorded in June (6/17; 35.3%). The children ranged in age from 10 to 25 months, with most of them in their second year of life (15/17, 88.2%). 

The majority of children were born at term (11/17; 64.7%) via vaginal delivery (10/17; 58.8%) with weights ranging from 2,270 to 3,950 g. Jaundice (5/17; 29.4%) and respiratory distress (2/17; 11.8%) were the most common complications.

The children who did not participate in the study (13) had an average birth weight of 3,346 grams, ranging from 2,970 to 4,180 grams. Only one child was born preterm (34 weeks), and all the others were born at more than 38 weeks of gestation. They possessed similar characteristics as those of the babies included in the study.

At the time of the home visits, the cephalic perimeters of the children ranged from 42 to 48 cm, which were all suitable for their age. The Z-scores ranged from above 1 standard deviation to above -2 standard deviation; therefore, they did not raise a microcephaly alert. All the evaluated children had adequate weights for their age, ranging from 10,100 to 15,200 g (Table 3). However, 2 children presented with lengths below -3 Z-score, which represented a very short length for age.

**TABLE 3:** Evaluation of the child at birth and after the home visit.

N	Sex	GA at	Weight at	Height	Cephalic	Thoracic	Baby age at time	HC	Z-score	Weight	Z-score	Height	Z-score	PT
º		birth	birth (grams)	(cm)	perimeter (cm)	perimeter (cm)	of evaluation (months)	(cm)		(grams)	(weight)	(cm)	(height)	(cm)
1	M	35	2790	46	34	30.5	10	47	Suitable	10100	Suitable	76	Suitable	40
2	M	37	3105	51	34.5	33	15	48	Suitable	11000	Suitable	78	Suitable	42
3	M	38	3295	47	32.5	31.5	16	46	Suitable	12800	Suitable	82	High	44
4	M	41	3750	51	37	36	18	45	Suitable	13100	Suitable	80	Suitable	44
5	F	39	3580	52	35	33	20	47,5	Suitable	10900	Suitable	84	Suitable	42
6	F	38	2760	47	34	31.5	20	48	Suitable	15000	Suitable	77	Suitable	44
7	F	37	2275	46	33	29	20	46	Suitable	15000	Suitable	78	Suitable	42
8	F	37	2270	42	34.5	30.5	20	46	Suitable	10600	Suitable	80	Suitable	42
9	F	38	3950	49	34	34	20	48	Suitable	12500	Suitable	84	Suitable	40
10	F	36	2634	49	35.5	30.5	21	47	Suitable	11250	Suitable	80	Low	42
11	M	40	3600	51	33.5	-	21	47	Suitable	11000	Suitable	79	Low	58
12	M	39	3264	51	35	33	21	47	Suitable	12500	Suitable	86	Suitable	48
13	M	40	3190	50	35	33	21	42	Suitable	10100	Suitable	75	Suitable	38
14	M	38	3536	48	34.5	34	22	46	Suitable	14600	Suitable	88	Suitable	46
15	F	37	3235	48	33.1	-	23	46	Suitable	12300	Suitable	86	Suitable	40
16	F	38	3140	48	35	32	24	47	Suitable	15200	Suitable	88	Suitable	46
17	M	36	2820	47	33		25	46	Suitable	12000	Suitable	79	Suitable	40

Legend: **GA:** Gestational age; **PT:** thoracic perimeter.

During childcare, 16/17 (94.1%) children had records of evaluation in the child's booklet. Five children had records of neurological development, and none had records of developmental changes. Furthermore, 7/17 (41.2%) did not undergo any kind of systematic medical monitoring. Regarding the evaluation of the developmental milestones, 15/17 (88.2%) presented with at least one delayed developmental milestone with respect to the standards for the age group. Among these children, 5/15 (33.3%) reached three developmental milestones, 5/15 (33.3%) reached two, and 5/15 (33.3%) reached only one (Table 4).

**TABLE 4:** Assessment of children’s developmental milestones according to the child's health booklet.

Nº	Age (months)	Sex	DNPM Evaluation	Milestones achieved	Show what they want	Put blocks in the box	Speaks a word	Walks without support	Uses spoon or fork	Constructed towers of two cubes	Speaks 3 words	Walks backwards	Undresses	Constructed towers with 3 cubes	Aims two figures	Kicks the ball	Dresses with supervision	Constructed towers of 6 cubes	Phrases with two words	Jumps with two feet
1	10	M	Alert	1	+	-	-	-	●	●	●	●	●	●	●	●	●	●	●	●
2	15	M	Normal	4	+	-	+	-	+	+	+	+	●	●	●	●	●	●	●	●
3	16	M	Alert	1	+	-	-	+	-	-	-	+	●	●	●	●	●	●	●	●
4	18	M	Alert	1	+	+	-	+	-	-	-	+	●	●	●	●	●	●	●	●
5	20	F	Alert	3	+	+	+	+	+	+	+	+	+	-	+	+	●	●	●	●
6	20	F	Alert	2	+	-	+	+	+	-	+	+	+	-	-	+	●	●	●	●
7	20	F	Alert	2	+	+	+	+	+	-	-	+	+	-	-	+	●	●	●	●
8	20	F	Alert	2	+	+	+	+	+	-	+	+	-	-	+	+	●	●	●	●
9	20	F	Alert	2	+	+	+	+	+	-	+	+	+	-	+	+	●	●	●	●
10	20	F	Normal	4	+	+	+	+	+	-	+	+	+	+	+	+	●	●	●	●
11	21	M	Alert	1	+	+	+	+	+	-	-	+	-	-	-	+	●	●	●	●
12	21	M	Alert	3	+	+	+	+	+	+	-	+	-	+	+	+	●	●	●	●
13	21	M	Alert	2	+	+	+	+	+	-	-	+	-	-	+	+	●	●	●	●
14	22	M	Alert	1	-	-	-	+	+	-	-	+	-	-	-	+	●	●	●	●
15	23	F	Alert	3	+	-	+	+	+	-	+	+	+	-	+	+	●	●	●	●
16	24	F	Alert	3	+	-	+	+	+	-	+	+	+	-	+	+	●	●	●	●
17	25	M	Alert	3	+	-	+	+	+	-	+	+	+	-	+	+	+	-	+	+

Key: ● Unevaluated milestones for that age. (+) Milestone achieved: (-) Milestone not achieved. DNPM: neuropsychomotor development.

## DISCUSSION

The present study describes a group of 17 children born without microcephaly but whose mothers had Zika during pregnancy. It is important to consider that because they were not diagnosed with microcephaly at birth, these children were not monitored according to the orientation protocol of the Ministry of Health for children with CZS. Therefore, they were not evaluated in specialized services, and, instead, follow-up was performed by a primary health care provider. Considering the high percentage of children in this study who had not achieved all the developmental milestones for age and sex, we believe that it is important to re-evaluate them, due to the possibility of presenting late changes, mainly in neurological development. This recommendation has already been made by the Ministry of Health^5^. Studies such as the one by Ventura et al. (2016) point out the need to evaluate all children with intra-uterine exposures, even if they are asymptomatic, since there is a possibility that they may present some manifestation of CZS^11^. This birth evaluation was not performed in most of the children in this study. The guidelines of the Ministry of Health of Brazil suggest that children exposed to Zika during pregnancy should be supported by a primary health care service^5^. The growth and development curves contained in the child's health handbook are important tools for assessing the child’s development and for monitoring any changes or delays. This booklet is a simple screening tool to be used by a health professional that serves for monitoring developmental changes. When faced with an “alert” result in the evaluation, the child should be referred to specialized services or undergo closer monitoring in the following consultations. 

In this study, there was no significant difference between the sexes, despite the inclusion of more male children; this was also observed by Einspieler et al*.* (2019) and Vargas et al*.* (2016)^11^ ^,^ ^12^. It is noteworthy that even in studies where there was a difference between the sexes, this difference was never considered important, nor was it statistically significant. In view of this finding, it is possible that there is no sex-specific predominance in asymptomatic children exposed to ZIKV during pregnancy. 

The first cases of microcephaly associated with ZIKV in the municipality of Fortaleza were recorded in October 2015, and the last confirmed case was in December 2016, with a peak in the number of births between December 2015 and January 2016^13^. Children in this series of cases were born at a different time period from that in which there were CZS cases. In the months of June and August 2016, the peak period of births of the children in our study, there were no confirmed cases of children with CZS in the city of Fortaleza, suggesting that the periods of occurrences of the events were different. Unfortunately, there is no record of viral isolation of the strains that circulated during these two periods to evaluate, for example, whether there was a difference or possibility of increased virulence in the outbreak period (December or January), which could explain the lack of symptomatic children in our study.

Most of the mothers of the children evaluated were young, white, in a stable relationship, who declared themselves as housewives, and had family incomes of just over one minimum wage. Carvalho-Sauer (2019) found different maternal characteristics in children with CZS in Bahia, where women who were brown, very young, single, and had low levels of schooling predominated^14^. Gonçalves et al. (2018) also found the same characteristics in the mothers of children with CZS: predominantly single women, housewives, and most with monthly incomes of up to one minimum wage^15^. The profiles of mothers with asymptomatic children differs from that of those who had children with microcephaly. Although these studies cover very small populations, their findings should not be disregarded. 

A rash was presented by all the mothers during pregnancy, and it was pruriginous in most of them. These symptoms predominated in the second and third trimesters of gestation, corroborating the findings of other studies involving children born without microcephaly^12^. Another study evaluating Zika-positive pregnant women also showed that 100% of mothers had a rash^16^. In children with CZS, infection predominantly occurred in the first trimester of gestation^17^. A study by France et al. (2016) evaluating children with ZIKV-associated microcephaly found that 61.4% of mothers had exanthema during the first trimester of pregnancy^18^. According to Brady et al. (2019), women infected with ZIKV in early gestation were 17 times more likely to have children with microcephaly^19^. This would justify the results of our study regarding the period of infection of the pregnant women, reinforcing the fact that a rash is an important clinical sign to be identified; however, it does not guarantee that the child will be born with CZS. This may vary, mainly depending on the trimester of gestation in which the symptoms appear, like in the case of other congenital syndromes.

A majority of the mothers in our study had not previously been vaccinated against yellow fever. Studies such as the one by Cavalcanti et al. (2016) raised the hypothesis that vaccination against yellow fever could reduce the risk of the baby presenting with microcephaly as a result of maternal infection with ZIKV^20^. Vicente et al. (2019) reinforced this hypothesis by testing the vaccine in mice and verifying that it provides strong protection to the fetus against ZIKV, resulting in lower mortality, lower viral load, and fewer neurological signs of ZIKV infection^21^. Unfortunately, this hypothesis could not be tested in our study because of the small number of vaccinated women and the limited sample size. 

All the children evaluated were full term with normal weight, similar to the findings of the study by Cardoso et al. (2018)^7^.

In most of the evaluated children, there were no records of imaging tests performed at birth to exclude brain alterations. Martins et al. (2018) found that all children without microcephaly who were evaluated had some alterations in CZS-compatible imaging tests^17^. Lindenet et al. (2018) evaluated 13 children without microcephaly and found that they all had central nervous system malformations based on brain computed tomography results^22^. Einspieler et al*.* (2019) also described this phenomenon in children without microcephaly, but in a smaller proportion^12^. In view of these findings, it would be important to evaluate all children exposed to ZIKV during pregnancy, even if they were born without microcephaly. We report that, currently, these children are not being evaluated through specialized examinations for the diagnosis or dismissal of CZS. 

As regards auditory assessment, only five children underwent the appropriate examination and one presented with alterations in the left ear. In children with microcephaly, the prevalence of hearing loss was 6.6%^23^. No child had a more specialized ophthalmological evaluation performed, such as fundoscopy, which prevents us from commenting on important limitations in vision. A study by Ventura et al. (2016) has already identified important ocular lesions in children without microcephaly^11^, which would justify this type of examination in children who were exposed to ZIKV during pregnancy. The Ministry of Health’s National Guidelines on Surveillance and Health Care in the National Public Health Emergency Guidelines (2017) already recommend retinal mapping in children who have positive serology for ZIKV and/or neurological changes related to ZIKV infection and/or whose mothers have a history of exanthematous disease during gestation^5^. We also highlight the low percentage of completion of the child's booklet regarding aspects related to neuropsychomotor development. This situation has already been reported in other scenarios. Moreover, even when the child's booklet was used, only over one-third recorded these conditions^24^ ^,^ ^25^ ^,^ ^26^.

Most of the evaluated children had cephalic perimeters, weights, and heights suitable for their ages. The same was observed in the study by Prata-Barbosa et al. (2019) in children born without microcephaly to mothers who had ZIKV during gestation^27^. This is likely because weight-stature gain is associated with the degree of neurological impairment in children with or without microcephaly, provided they have some type of neurological impairment. Even so, it is recommended to monitor the child’s development during the first few years of life, with the aim of identifying a slowdown in the increase in HCs, weights, and heights as early as possible. 

It is also worth noting that almost all the children evaluated were “alert” to the developmental milestones. This has already been reported in other cases, but it is not common when "normal" children are evaluated^7^ ^,^ ^12^ ^,^ ^28^. These findings suggest that children who were exposed to ZIKV during gestation, but were born asymptomatic, are at an increased risk for abnormal neurological development. It is necessary to investigate whether, in the future, even with appropriate treatment, these children will reach age-appropriate milestones. 

It is noteworthy that despite residing in a large Brazilian capital city, most children had not received adequate developmental monitoring and the mothers had no information about that need. Based on the evaluation of the field researcher, we highlight the perception that many children presented with delayed speech and a marked degree of agitation, as well as a suspicion of autism in one child - all characteristics that have already been reported by other authors^7^ ^,^ ^28^ ^,^ ^29^.

A limitation that we cannot disregard was the small number of children evaluated, which prevented us from extrapolating the findings to the entire population of children who were exposed to ZIKV but did not present with microcephaly at birth. However, it is important to point out that other small case series studies also show similar results, highlighting the importance of the developmental evaluation of these children. Another important limitation was the mothers' denial of the possibility of their children having any developmental changes related to ZIKV. After all the stress they had suffered during pregnancy, they were relieved at birth to see a baby without microcephaly, who was apparently normal. To discover that the child might have some late manifestation caused concern, and some mothers even refused to participate in the research or may have omitted providing some important information.
